# mTORC1 suppression by Trp53 mutation drives resistance to immune checkpoint blockade

**DOI:** 10.1038/s41419-026-09067-4

**Published:** 2026-07-07

**Authors:** Alireza Labani-Motlagh, Yang Li, David Shihong Gao, Eunseuk Lee, Vincent Chen, Lixian Yi, Fei Gao, Runzi Sun, Junchi Xu, Jason Shoush, Zoi Kykrou, Minxin Liang, Xinghua Lu, Binfeng Lu

**Affiliations:** 1 Center for Discovery and Innovation, Hackensack Meridian Health, Nutley, NJ USA; 2 Department of Immunology, University of Pittsburgh School of Medicine, Pittsburgh, PA USA; 3 Department of Medical Sciences, Hackensack Meridian School of Medicine, Nutley, NJ USA; 4 Biomedical Informatics Training Program, University of Pittsburgh School of Medicine, Pittsburgh, PA USA; 5 Georgetown Lombardi Comprehensive Cancer Center, Washington, DC USA

**Keywords:** Tumour immunology, Tumour-suppressor proteins

## Abstract

p53 is a critical tumor suppressor gene that inhibits cancer development by regulating cell cycle arrest, apoptosis, DNA repair, and metabolism. However, recent studies examining *TP53* mutations in cancer immunotherapy have yielded inconsistent results, likely due to differences in tumor mutational burden (TMB) and the context-dependent roles of specific p53 mutants. In this study, we assessed the function of G242V and S258I *Trp53* mutations in MC38 cells in the context of immunotherapy by generating *Trp53* deletion and observed significantly enhanced responses to anti-PD-1 therapy. *Trp53*-null tumors showed increased CD8^+^ T cell infiltration and clonal expansion, along with reduced regulatory T (Treg) cells. Mechanistically, *Trp53* deletion downregulated mTORC1 inhibitor genes, leading to elevated mTORC1 signaling and diminished autophagy, which sensitized tumor cells to IFN-γ and TNF-α-induced apoptosis. Besides mouse cells, we confirmed the human p53 mutants regulate the same sets of mTORC1 inhibitor genes in a human colorectal cancer cell line. Our findings demonstrate that certain p53 mutants, despite losing other canonical functions, retain wild type p53’s ability to suppress mTORC1 and enhance autophagy, thereby inhibiting responses to immunotherapy.

## Introduction

The p53 tumor suppressor protein, encoded by the *TP53* gene, is a critical regulator of cellular processes and plays a fundamental role in cancer prevention. Often referred to as the “guardian of the genome,” p53 is involved in multiple central cellular functions, including transcription, DNA repair, genomic stability, cell cycle control, and apoptosis [1, 2]. The importance of p53 in tumor suppression is underscored by its frequent inactivation in human cancers, with mutations in *TP53* observed in more than half of all sporadic tumors [2, 3]. While the classical functions of p53 in cell cycle arrest, senescence, apoptosis, and metabolism have been well-established, recent research has unveiled its involvement in regulating tumor immune responses [2, 4].

Recent studies on the role of *TP53* mutations in cancer immunotherapy have yielded complex and sometimes contradictory results. p53 activation in cancer cells can induce immune activating signals such as tumor necrosis factor-related apoptosis-inducing ligand (TRAIL), death receptor 5 (DR5), toll-like receptors (TLRs), and the cyclic GMP–AMP synthase (cGAS)–stimulator of IFN genes (STING) pathway [5]. P53 mutants were shown to suppress antitumor immunity by interfering with the function of the cytoplasmic DNA sensing machinery, cGAS-STING-TBK1-IRF3 [6]. Conversely, the activation of p53 in cancer cells can suppress immunity via increasing PDL1 levels [5]. In addition, *TP53* mutations have been found to be associated with increased lymphocytes in a large-scale pan-cancer genomics and transcriptomic study [7]. In human non-small cell lung cancer (NSCLC) patients, *TP53* mutations are significantly associated with levels of immune checkpoint molecules, activated T-effector genes, and IFNG signature genes [8, 9]. NSCLC patients with co-occurring *TP53/KRAS* mutations showed improved clinical benefits after treatment with PD-1 inhibitors [9]. In lung cancer patients treated with PD-1 inhibitors, *TP53* mutations have been associated with better overall response rates and overall survival [10–12]. For relapsed/refractory (R/R) acute myeloid leukemia (AML) patients treated with Flotetuzumab, an investigational CD123xCD3 bispecific dual-affinity retargeting antibody (DART) molecule, higher expression of immune markers such as IFNG, FOXP3, and immune checkpoints was observed in primary bone marrow samples with *TP53* mutations when compared to those with wild-type *TP53*. Moreover, *TP53* mutations and deletions are associated with clinical response to Flotetuzumab immunotherapy in AML [13]. In a neoadjuvant setting, a recent phase 2 clinical trial examined the combination of anti-CTLA4 and PD-1 monoclonal antibodies (mAbs) for operable NSCLC. The trial revealed that resected tumors with *TP53* alterations exhibited a higher median pathological regression compared to tumors with wild-type P53 [14]. The enhanced immune response observed against *TP53*-mutated cancers is thought to be attributed to an increased total mutational burden (TMB). This hypothesis aligns with the well-established role of p53 as a critical guardian of genomic stability. The discrepancies in these findings highlight the nuanced impact of *TP53* mutations on immunotherapy response, which likely depends on factors such as the specific mutation type, cancer type, and broader genetic context such as TMB. This complexity underscores the need for further research to fully elucidate the relationship between *TP53* mutations and immunotherapy outcomes.

To investigate the role of p53 mutants in immunotherapy, we utilized MC38, a mouse colorectal cancer cell line known for its responsiveness to immunotherapy and reported to harbor *Trp53* mutations [15]. Initially, we confirmed the presence of *Trp53* mutations in the parental MC38 cells. We then explored the tumor-intrinsic effects of p53 mutants by generating *Trp53*-deficient MC38 cells and evaluating their impact on the immunogenicity of the cell line. Additionally, we compared the influence of mutant p53 and *Trp53* deletion on the efficacy of PD-1 blockade immunotherapy in a murine tumor immune therapy model. To gain mechanistic insights into the immune-modulatory effects of mutant p53 in vivo, we performed single-cell RNA sequencing (scRNA-seq) and immunological analyses of both parental MC38 and *Trp53*-deficient MC38 tumor cells, as well as their associated TMEs. Lastly, we elucidated the molecular mechanisms by which p53 mutants regulate antitumor immunity in both mouse and human colorectal cancer cells.

## Results

### The *Trp53* mutations conferred resistance to cancer immunotherapy

To investigate the role of p53 in immune checkpoint inhibitor (ICI) therapy, we utilized MC38, a mouse colorectal cancer model known for its responsiveness to ICI treatment. Previous studies reported that MC38 harbors two mutated alleles of the *Trp53* gene [15]. We characterized the *Trp53* genomic region in MC38 cells and confirmed the presence of two mutations: G242V and S258I. Notably, the G242V mutation corresponds to the human *TP53* hotspot mutation G245S, frequently observed in human cancers. Additionally, the *TP53* G245S mutation is a missense mutation known to disrupt the structure of p53 and its ability to bind DNA [16]. The S258I mutation, on the other hand, is located at a splice site. This mutation has been shown to result in aberrant splicing, effectively creating a null allele of *Trp53* [17]. The nature of these mutations has been confirmed by our genomic and RNA sequencing (data not shown).

**Fig. 1:**
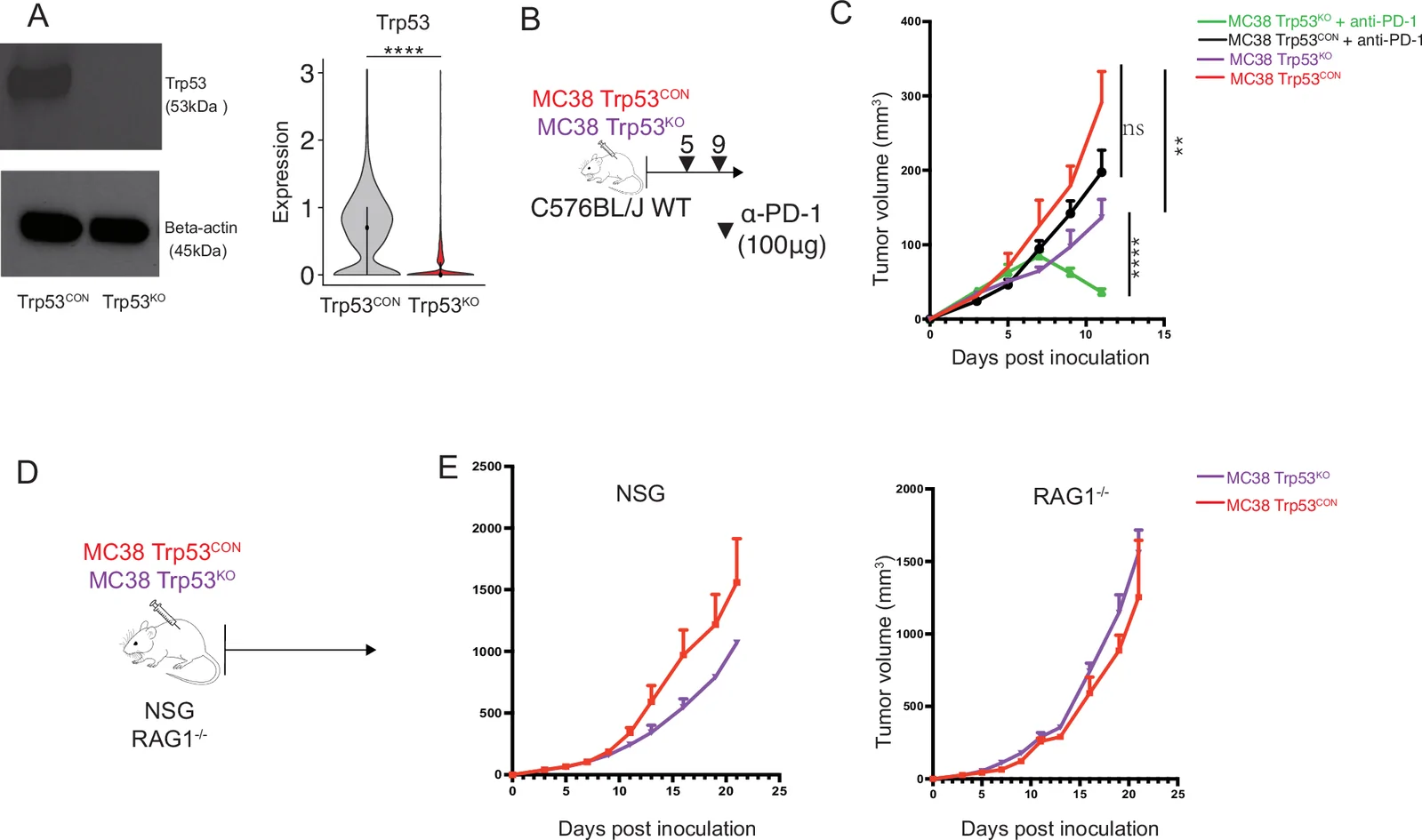
*Trp53* deletion in tumor cells increases response to cancer immunotherapy. **A** *Trp53* protein and mRNA levels in *Trp53* “knock-out” (*Trp53*^KO^) and control (*Trp53*^CON^) MC38 tumor cells were assessed using Western blot analysis of in vitro cultured cells and scRNA-seq on isolated tumors, respectively. **B** Schematic drawing of anti-PD1 monoclonal antibody (mAb) treatment in *Trp53*^CON^ and *Trp53*^KO^ tumors. **C** *Trp53*^CON^ and *Trp53*^KO^ tumor cells were inoculated into C57BL/6 J mice (*n* = 5), treated with anti-PD-1 therapy, and tumor growth was measured. **D**–**E** *Trp53*^CON^ and *Trp53*^KO^ tumor cells were inoculated into NSG or RAG1-/- mice (*n* = 5), and tumor growth was measured. **** *p* < 0.0001 ** *p* < 0.01, two-way ANOVA.

We definitively tested whether and how mutant p53 affects the efficacy of ICI immunotherapy using a loss-of-function approach in the transplant mouse model of MC38 colon adenocarcinoma. Using CRISPR-Cas9, we targeted for deletion a region of exon 4 in the *Trp53* gene that encodes in the proline-rich domain and a part of the DNA-binding domain. Deletion of this region resulted in the elimination of p53 protein expression in vitro and *Trp53* mRNA expression in vivo (Fig. 1A). These data show that we have generated a *Trp53*-null MC38 cell line, which we hereafter term *Trp53*^KO^ cells, along with their unedited controls, *Trp53*^CON^ cells.

We next tested the response of *Trp53*^CON^ and *Trp53*^KO^ cells to anti-PD-1 therapy in vivo (Fig. 1B). As early as day 9 – a time point at which anti-PD-1 therapy does not yet affect the growth of WT tumors – treated *Trp53*^KO^ tumors already had a significantly smaller volume compared to both untreated and treated *Trp53*^CON^ MC38 tumors. These differences remained at later time points, with 1/3 of *Trp53*^KO^ tumors undergoing complete remission (Fig. 1C). Interestingly, untreated *Trp53*^KO^ tumors also grew slower than untreated and treated *Trp53*^CON^ MC38 tumors, though faster than their treated counterparts. The results were repeated with another independent *Trp53*^KO^ MC38 clone (data not shown). Our results provide functional evidence that the p53 mutant in MC38 cells inhibits the efficacy of cancer immunotherapy.

We next determined whether tumor cell-intrinsic or -extrinsic immune response changes were responsible for the reduction in tumor growth. *Trp53*^CON^ and *Trp53*^KO^ MC38 cells were inoculated into immunodeficient RAG1^-/-^ (lack B and T cells) and NSG (lack B, T, and NK cells) mice (Fig. 1D). We found no difference in tumor growth between the *Trp53*^CON^ and *Trp53*^KO^ cells in either immunodeficient mice strains (Fig. 1E). These data show that alterations to the immune response, and not cell-intrinsic changes induced by *Trp53* deficiency in tumor cells, are required for the reduction of tumor growth and improved efficacy of checkpoint immunotherapy.

### Characterization of the immune responses in *Trp53*^CON^ and *Trp53*^KO^ tumors during anti-PD-1 therapy

**Fig. 2:**
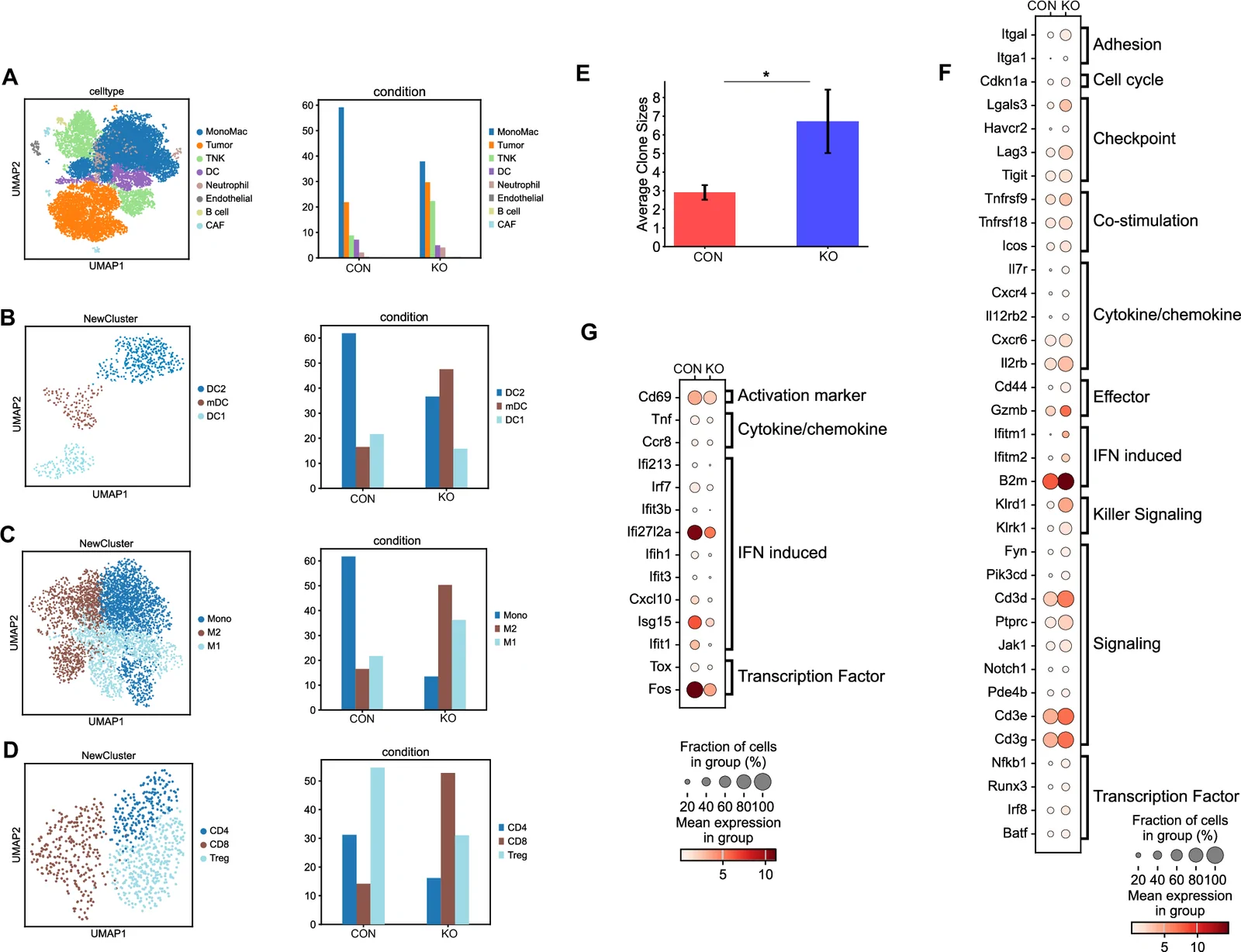
*Trp53* deletion led to enhanced adaptive antitumor immune responses in the TME. **A** *Trp53*^CON^ (CON) and *Trp53*^KO^ (KO) tumors were treated with anti-PD1 mAb. Ten days post-tumor cell inoculation, paired scRNAseq and scTCRseq were performed. The left panel shows a Uniform Manifold Approximation and Projection (UMAP) plot illustrating the diverse cell populations within the TME. The right panel presents the relative proportions of these identified cell types. **B** The left panel shows a UMAP plot illustrating the DC subsets. The right panel presents the relative proportions of DC subsets. **C** The left panel shows a UMAP plot illustrating the monocyte and macrophage subsets. The right panel presents the relative proportions of monocyte and macrophage. **D** The left panel shows a UMAP plot illustrating the T cell lineages. The right panel presents the relative proportions of T cell lineages. **E** Clonal expansion of CD8^+^ T cells in the TME. **F** Dot plot showing genes with elevated expression in CD8^+^ T cells from *Trp53*^KO^ tumors, for comparison with *Trp53*^CON^. **G** Dot plot showing genes with elevated expression in CD8^+^ T cells from *Trp53*^CON^ tumors. * *p* < 0.05, by Student’s *t*-test.

We next characterized how the *Trp53* deletion in MC38 tumor cells alters the immune response. To this end, we performed single-cell RNA and paired single-cell TCR sequencing (scRNAseq and scTCRseq) of anti-PD-1 treated *Trp53*^CON^ and *Trp53*^KO^ tumors on day 11 (Fig. 2A). We observed a decrease in monocytic myeloid-derived suppressor cells (mMDSCs) and a slight increase in neutrophils in *Trp53*^KO^ tumors compared to *Trp53*^CON^ tumors. Notably, TNK cells, encompassing all T cells, NK cells, and ILCs, were predominantly increased in *Trp53*^KO^ tumors relative to *Trp53*^CON^ tumors (Fig. 2A). Although there is no striking difference in the total DC population, both the cDC1 and cDC2 populations decreased, while mature DCs increased in *Trp53*^KO^ tumors, suggesting an increased DC maturation in these tumors (Fig. 2B). In the monocyte/macrophage compartment, we observed an increase in macrophage 1 (Mac1) and macrophage 2 (Mac2) populations, suggesting an enhanced monocyte-to-macrophage differentiation program (Fig. 2C). Within the T cell population, we found that CD8^+^ T cells increased in *Trp53*^KO^ tumors while Treg cells decreased (Fig. 2D). Paired scTCR-seq enabled us to analyze clonal expansion in T cells. We found that the average clonal size of expanded clones was significantly larger in CD8⁺ T cells from *Trp53*^KO^ tumors than those from *Trp53*^CON^ tumors (Fig. 2E). We also analyzed differential gene expression in CD8⁺ T cells from *Trp53*^KO^ and *Trp53*^CON^ tumors. CD8⁺ T cells from *Trp53*^KO^ tumors exhibited higher expression levels of checkpoint molecules, including *Lag3*, *Tim3*, and *Tigit*, as well as costimulatory molecules such as *4-1bb*, *Gitr*, and *Icos*. Additionally, they expressed higher levels of chemokine receptors (*Cxcr6*), effector molecules (*Gzmb*), signaling molecules involved in the TCR complex, and transcription factors that drive effector T cell function, including *Nfkb1*, *Runx3*, *Irf8*, and *Batf* (Fig. 2F). In contrast, CD8⁺ T cells from *Trp53*^CON^ tumors predominantly expressed early activation markers such as *Cd69* and *Fos*, along with chronic inflammation-associated genes, particularly interferon (IFN)-induced genes (Fig. 2G). Collectively, our analysis reveals that CD8⁺ T cells in *Trp53*^KO^ tumors undergo greater clonal expansion and exhibit a more activated phenotype, which aligns with the increased susceptibility of *Trp53*^KO^ tumors to anit-PD1 ICI treatment.

**Fig. 3:**
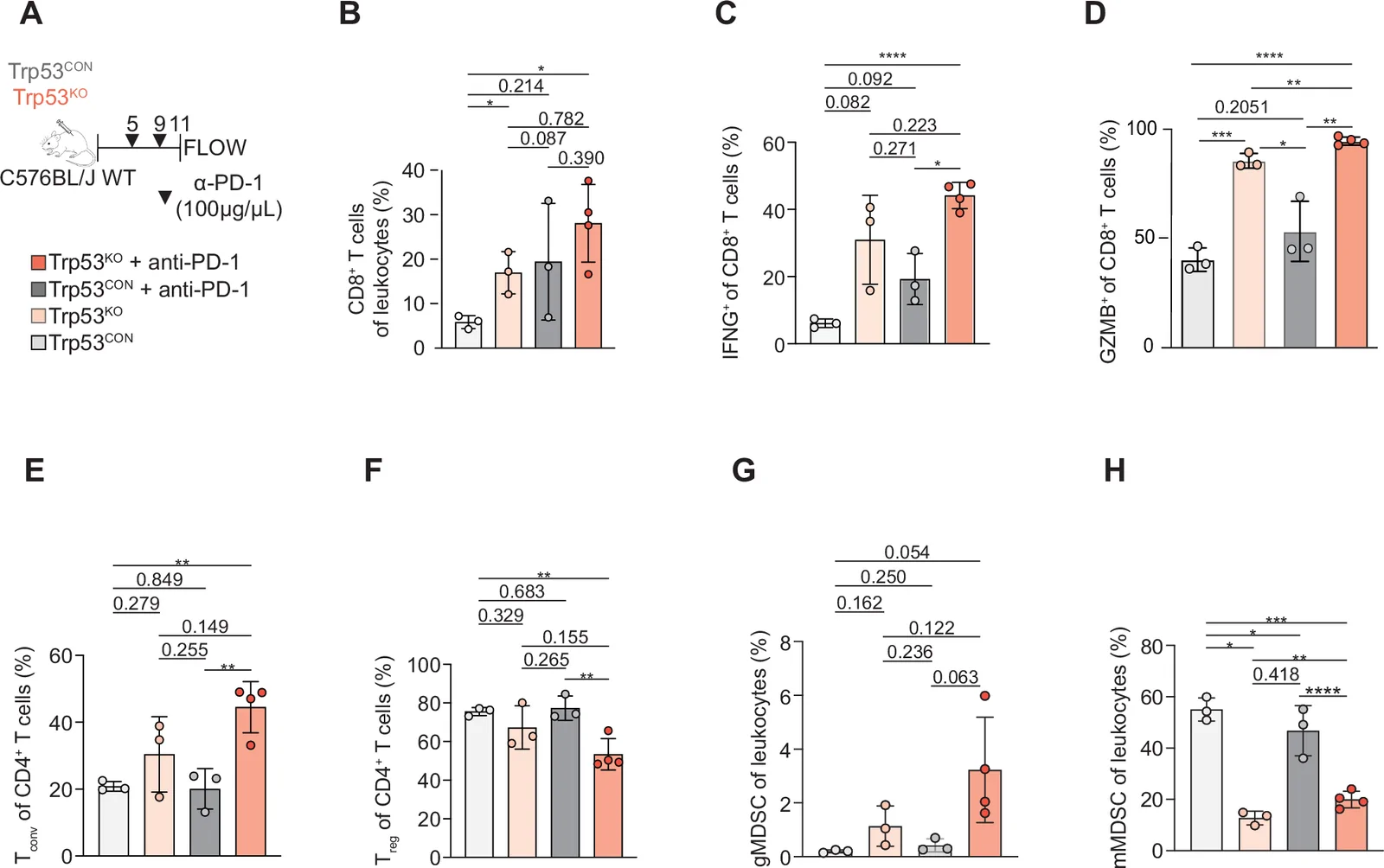
Loss of *Trp53* in tumor cells remodels the immune compartment during anti-PD-1 therapy. **A** Anti-PD1 mAb treatment was administered to *Trp53*^CON^ and *Trp53*^KO^ tumors, as depicted in the schematic. Ten days after tumor inoculation, tumors were harvested, and single-cell suspensions were prepared for flow cytometry analysis. **B** Percentages of CD8^+^ TIL in live lymphocytes are shown. **C** Percentages of IFN-γ-producing CD8^+^ TIL in CD8^+^ T cells. **D** Percentages of GZMB-producing CD8^+^ TIL in CD8^+^ T cells. **E** Percentages of conventional CD4^+^ T cells among total CD4^+^ TILs are shown. **F** Percentages of Tregs in CD4^+^ T cells. **G** Percentages of granulocytic myeloid-derived suppressor cells (gMDSC). **H** Percentages of monocytic myeloid-derived suppressor cells (mMDSC). **** *p* < 0.0001, *** *p* < 0.005, ** *p* < 0.01, * *p* < 0.05, two-way ANOVA.

We validated our characterization of the immune response by performing multicolor flow cytometry (Fig. 3A). We found that treated *Trp53*^KO^ tumors had increased CD8^+^ T cells that expressed an intense type 1 signature of IFN-γ and GzmB, compared to both untreated and treated *Trp53*^CON^ tumors (Fig. 3B–D, Supplementary Fig. 1). In addition, we also observed an increase in conventional CD4^+^ T cells and a decrease in Treg cells in the treated *Trp53*^KO^ tumors compared to both untreated and treated *Trp53*^CON^ tumors (Fig. 3E, F). Additionally, we observed a decrease in mMDSCs and a trend toward an increase in gMDSCs in *Trp53*^KO^ tumors compared to *Trp53*^CON^ tumors (Fig. 3G, H, Supplementary Fig. 2). Our profiling of the immune response consistently showed that *Trp53* deletion in tumor cells and anti-PD-1 therapy drastically alter the immune response in vivo, primarily augmenting T cell responses.

### *Trp53* deletion leads to altered oncogenic programs and increased immune signature genes in tumor cells

**Fig. 4:**
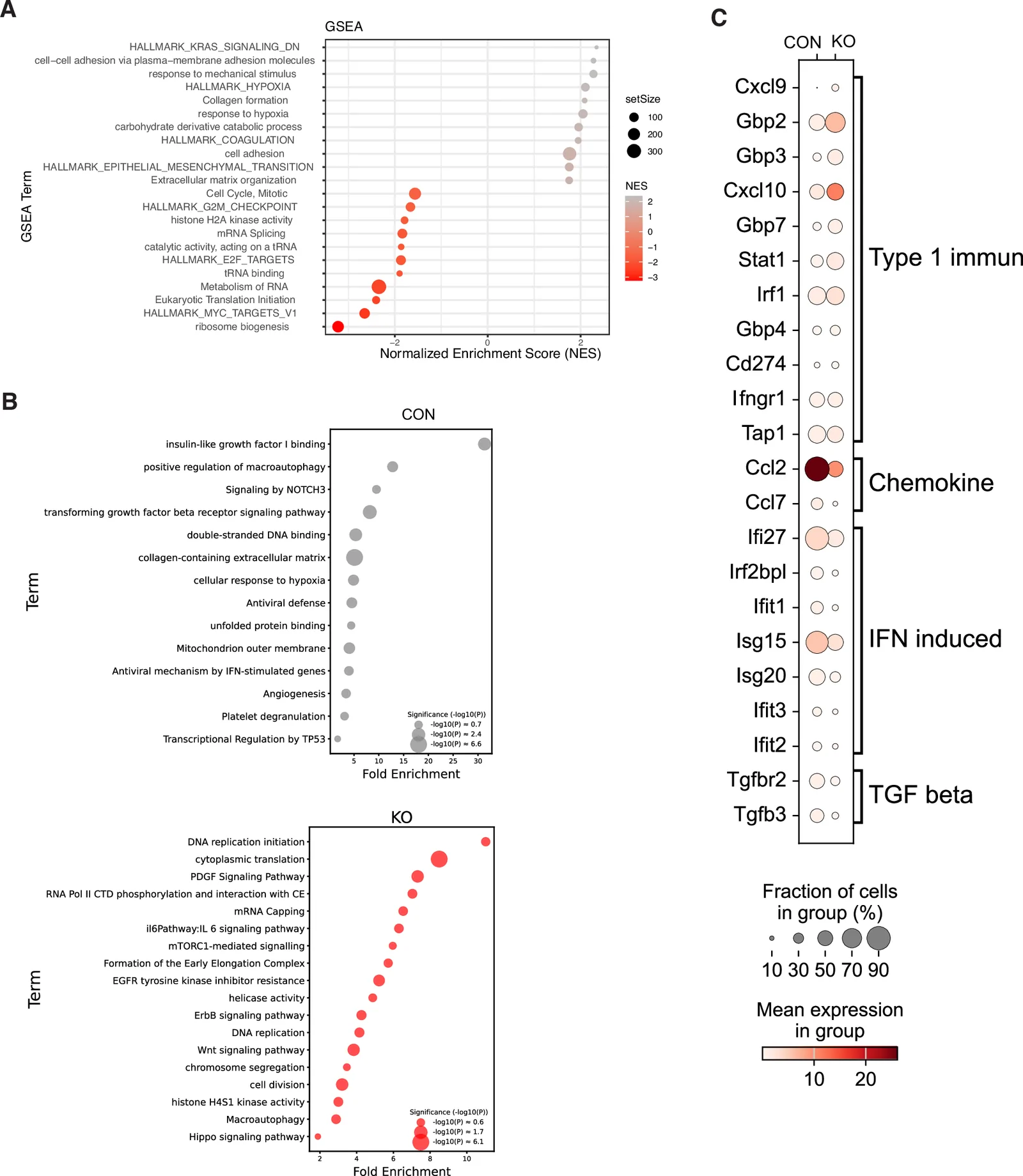
*Trp53* Deletion Alters Tumor Metabolism and Immunogenicity. Tumor cell counts from total tumor scRNAseqs were analyzed. **A** Gene Set Enrichment Analysis (GSEA) illustrating enriched pathways in *Trp53*^CON^ (CON, grey) and *Trp53*^KO^ (KO, red) tumor cells. **B** DAVID functional annotation analysis showing enriched pathways in *Trp53*^CON^ (grey) and *Trp53*^KO^ (red) tumor cells. **C** Dot plots depicting differentially expressed immune-related genes in *Trp53*^CON^ and *Trp53*^KO^ tumor cells.

The alteration of tumor growth prompted us to investigate how *Trp53* deletion affects tumor cells during cancer immunotherapy compared with *Trp53*^CON^ MC38 in vivo. We conducted a comprehensive analysis of differentially expressed genes (DEGs) in *Trp53*^CON^ and *Trp53*^KO^ MC38 cells in the scRNAseq data. Gene set enrichment analysis (GSEA) using gene ontology, hallmark, and reactome datasets showed that *Trp53*^CON^ cells exhibited upregulation of processes linked to epithelial-mesenchymal transition (EMT) and hypoxia, such as extracellular matrix organization, cell adhesion, coagulation, hypoxia response, collagen formation, and KRAS signaling downregulation (Fig. 4A and Supplementary Table 1).

In addition, we utilized the Database for Annotation, Visualization, and Integrated Discovery (DAVID) [18, 19] to functionally annotate the pathways, focusing on cancer cell-intrinsic programs that were differentially regulated based on the identified DEGs (Fig. 4B and Supplementary Table 2). The upregulated pathways in *Trp53*^CON^ MC38 cells can be categorized into transcriptional regulation by TP53, stromal responses, anti-viral responses, stress responses, Wnt signaling, and autophagy/metabolic regulation. The “Transcriptional Regulation by *Trp53*” pathway was significantly upregulated, reflecting residual canonical P53 function in MC38 cells, with genes such as *Btg2*, *Sesn3*, *Ubb*, *Zfp385a*, *Trp53*, *Ddit4*, *Tnks1bp1*, and *Cox6a2*. Stromal pathways were also prominently activated on *Trp53*^CON^ MC38 cells, including angiogenesis (*Acvrl1, Xbp1, Ecscsr, Fn1, Hspg2, Rhob, Eng*), collagen-containing extracellular matrix (*Scara3, Sparc, Fn1, Lamc1, Col1a1, Thbs2, Col5a1, Col5a2*, and *Serpinh1*), platelet degranulation (*Islr, Sparc, Fn1, Aldoa*, and *Clu*), signaling by Notch3 (*Ncstn, Ubb*, and *Wwp2*), and the TGF-beta receptor signaling signature (*Acvrl1, Jun, Cited1, Trp53*, and *Ccl2*), emphasizing tissue remodeling and immune modulation by the mutant p53. Anti-viral mechanisms were significantly upregulated, with pathways like antiviral defense (*Mavs, Apobec3, Rsad2, Ddit4, Isg15*, and *Ifit1*), double-stranded DNA binding (*Nr5a1, Egr1, Jun, Aim2, Jund, Fosb*, and *Fos*), IFN-stimulated genes (*Mavs, Isg15, Usp18*, and *Hspa1b*), and RNA binding (*Apobec3, Rbpms, Msi2*, and *Ifit1*) collectively indicating heightened nucleic acid sensing and antiviral responses in *Trp53*^CON^ MC38 cells. Stress pathways, such as unfolded protein binding (*Serpinh1, Clu, Cryab*, and *Hspa1b*) and cellular senescence (*Jun, Ubb, Trp53*, and *Fos*), reflect adaptations to cellular damage. The Wnt signaling pathway exhibited mixed regulation, with genes promoting Wnt signaling (*Rspo3, Rspo4, Wls, Csnk2a1, Csnk2a2*, and *Ccnd1*) and genes inhibiting it (*Hic1, Tle5*). Autophagy and metabolic pathways were highly induced, including mitochondrial function (*Cyb5b, Pink1, Mavs, Ubb, Bnip3, Ddit4*, and *Mgarp*), cellular response to hypoxia (*Pink1, Bnip3, Trp53*, and *Hif1a*), positive regulation of macroautophagy (*Pink1, Sesn3, Bnip3*, and *Hif1a*), and insulin-like growth factor I binding (*Igfbp5, Igfbp4*, and *Igfbp6*), highlighting mitochondrial dynamics, hypoxia adaptation, and enhanced autophagic processes. Together, these results reveal a complex response network involving transcriptional regulation, stromal remodeling, immune defense, stress adaptation, and metabolic reprogramming.

In *Trp53*^KO^ cells, GSEA analysis revealed that pathways associated with increased protein synthesis and proliferation were significantly upregulated, including ribosome biogenesis, MYC targets, eukaryotic translation initiation, RNA metabolism, E2F targets, mRNA splicing, and cell cycle processes (Fig. 4A and Supplementary Table 1). DAVID analysis showed a similar pattern of changes (Fig. 4B and Supplementary Table 2). The upregulated pathways in *Trp53*^KO^ MC38 cells span multiple major categories, reflecting diverse biological processes (Fig. 4B). Tumor promotion signaling pathways were also prominent, including “EGFR tyrosine kinase inhibitor resistance,” “ErbB signaling,” “Hippo signaling,” and multiple “Wnt signaling” pathways. These are driven by genes such as *Nras, Plcγ2, Prkca, Wnt5a, Sox9*, and *Csnk1e*, which regulate cell proliferation, differentiation, and oncogenic signaling. Angiogenesis-related pathways such as “Angiogenesis” and “PDGF signaling” were highlighted, with genes like *Pdgfa, Prkca*, and *Vegfa* indicating enhanced vascularization and extracellular matrix remodeling. In the mTOR category, pathways such as “mTOR signaling,” “PI3K-Akt signaling,” and “Amino acid regulation of mTORC1” were enriched, involving genes like *Nras, Lamtor3, Prkca*, and *Eif4e*, reflecting metabolic regulation and cellular growth. Autophagy pathways, including “Macroautophagy” and “Cellular response to starvation,” were driven by genes like *Gabarapl2, Lamtor3*, and *Tomm20*, emphasizing cellular recycling and stress responses. Protein translation promotion was observed through pathways such as “rRNA processing,” “cytoplasmic translation,” “mRNA processing,” and “Eukaryotic translation initiation,” involving genes like *Rpl31, Rpl34, Rps27*, and *Eif4e*. These pathways highlight robust translational activity. Gene transcription regulation pathways, including “Transcription by RNA polymerase II,” “RNA Pol II CTD phosphorylation,” and “Transcription initiation and promoter clearance,” involved genes such as *Polr2c, Gtf2h3*, and *Gtf2h5*. Lastly, DNA replication pathways, such as “Cell Cycle,” “DNA Replication,” “Mitotic Prometaphase,” and “Chromosome segregation,” featured genes like *Cdt1, Mcm3, Cdk1*, and *Cenpe*, reflecting cell division and genomic stability. Together, these pathways suggest enhanced mTOR signaling, reduced autophagy, and metabolic alterations that collectively promote cancer cell proliferation in *Trp53*^KO^ MC38 cells.

In addition to cancer cell gene programs, we analyzed immune gene programs differentially expressed in *Trp53*^CON^ and *Trp53*^KO^ MC38 cells in vivo. Notably, the type 1 immune response program, which includes genes such as *Cxcl9, Gbp2, Gbp3, Cxcl10, Gbp7, Stat1, Irf1, Gbp4*, and *Cd274*, was significantly enriched (Fig. 4C). In contrast, *Trp53*^CON^ cells exhibited higher expression of chemokines that attract myeloid cells, enhanced interferon signatures, and increased levels of TGF-β–related genes (Fig. 4C). These findings align with the observation that *Trp53*^KO^ MC38 tumors exhibit increased sensitivity to PD-1 blockade.

### p53 deficiency in MC38 cells increased sensitivity to TNF-α/IFN-γ-induced cancer cell death

**Fig. 5:**
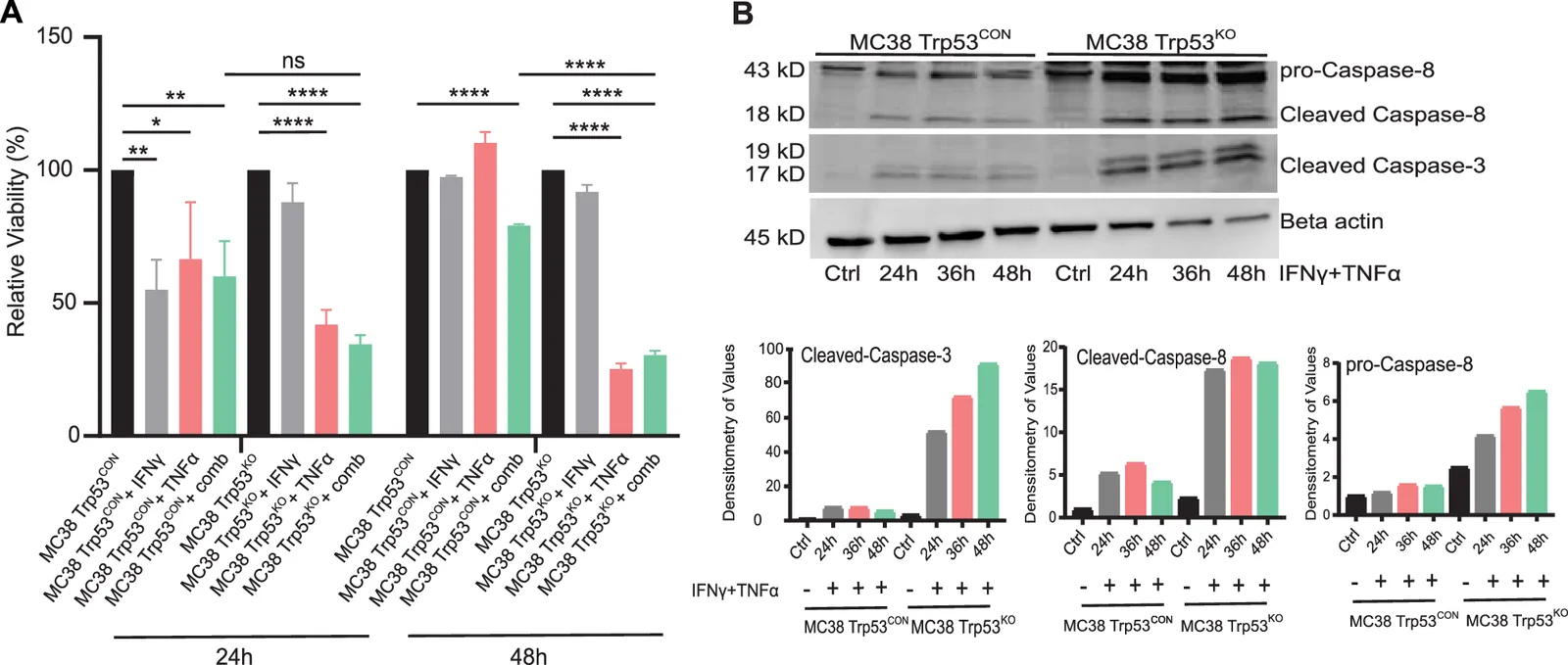
*Trp53* deletion led to increased cytokine-induced cell death. **A** Induction of cell death in *Trp53*^CON^ and *Trp53*^KO^ MC38 clones after 24 and 48 h of treatment with IFNγ, TNFα, or their combined application. Statistical significance was assessed using two-way ANOVA (*p < 0.05, **p < 0.01, ****p < 0.0001, ns = not significant, *n* = 3). **B** Changes in pro- and cleaved caspase-8 protein and cleaved caspase-3 levels in *Trp53*^CON^ and *Trp53*^KO^ MC38 cells following 24, 36, and 48 h of treatment with combined murine IFNγ and TNFα, or in untreated control cells. β-actin was used as a loading control. The lower panel displays densitometric quantification of the Western blot bands. Ctrl untreated control.

CD8^+^ T cells promote antitumor immunity through several mechanisms, including cytokine-triggered cell death by releasing inflammatory cytokines such as IFN-**γ** and tumor necrosis factor alpha (TNF-α), as well as perforin-dependent tumor cell killing [20]. Several recent studies showed that resistance to IFN-**γ** and TNF-α-mediated cancer cell death is a major mechanism of immune evasion [21–24]. Because we found that *Trp53* deletion in MC38 cells led to increased sensitivity to PD-1-blockade immunotherapy in vivo, we aimed to determine whether the p53 mutant regulates IFN-**γ** and TNF-α-induced cancer cell death in vitro. To this end, we incubated MC38 (p53^CON^ control clones) and *Trp53*^KO^-MC38 cells (two independently generated clones #6 and #16) with IFN-**γ**, TNF-α, or IFN-**γ** plus TNF-α. The combination of IFN-**γ** and TNF-α reduced live control MC38 (p53^MUT^) cells (Fig. 5A). Further analysis demonstrated an increase in apoptosis, as determined by Western blot of active caspase 3 and caspase 8, in *Trp53*^KO^ MC38 cells treated with IFN-**γ** and TNF-α when compared to control *Trp53*^CON^ MC38 cells (Fig. 5B). Notably, the protein level of pro-Caspase-8 was also increased in *Trp53*^KO^ MC38 cells compared to *Trp53*^CON^ MC38 cells (Fig. 5B). These data indicate that mutant p53 inhibits IFN-**γ** and TNF-α-induced tumor cell death.

### *Trp53* deletion in MC38 cells increased mTOR activation and diminished autophagy

**Fig. 6:**
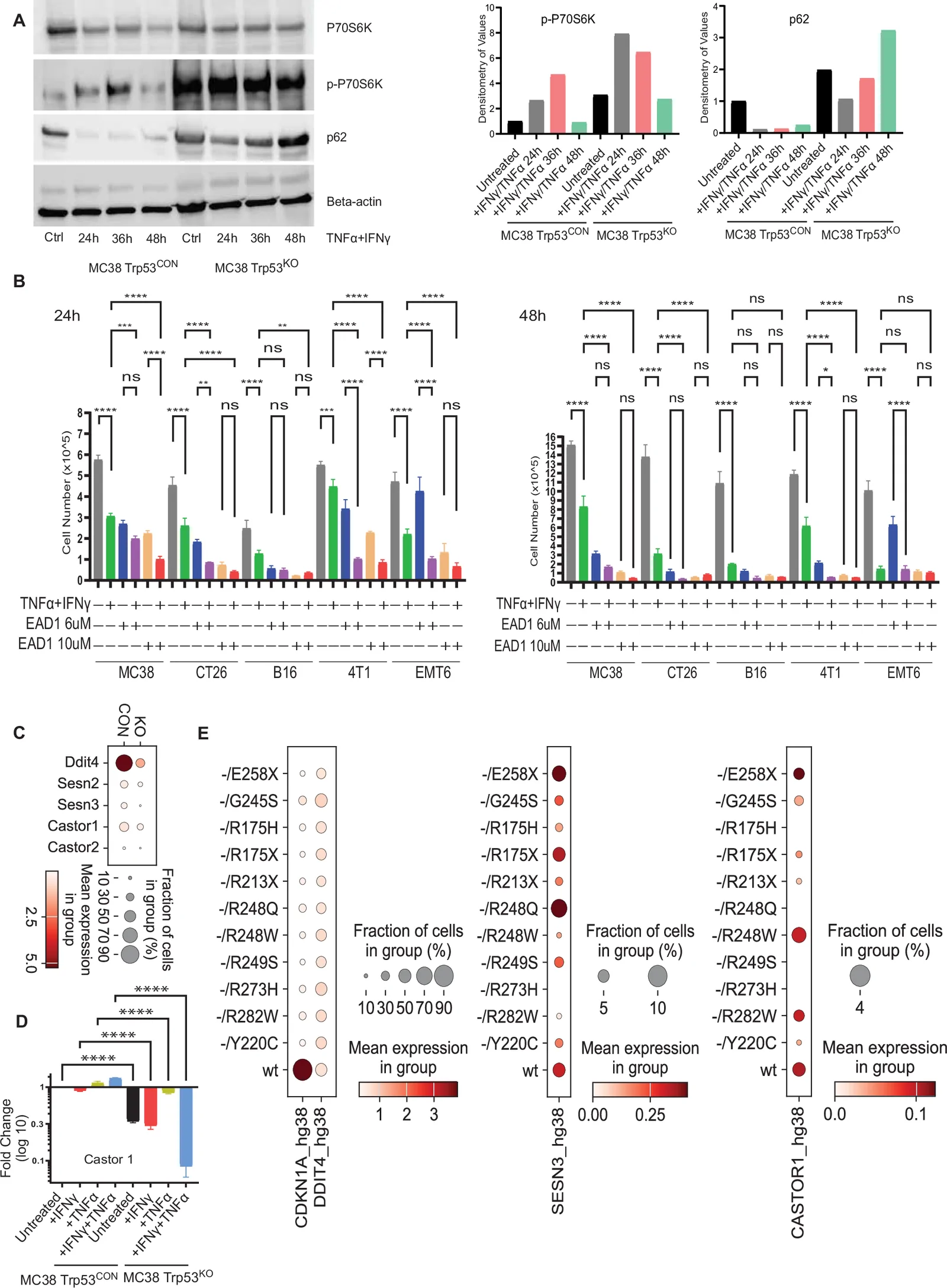
*Trp53* deletion led to increased mTORC1 activation and reduced autophagy. **A** Changes in phosphorylated p70S6K and p62 protein expression in *Trp53*^CON^ and *Trp53*^KO^ MC38 cells following 24, 36, and 48 h of treatment with murine IFNγ and TNFα. β-actin was used as a loading control. The lower panel displays densitometric quantification of the Western blot bands. Ctrl = untreated control. **B** Assessment of autophagy inhibition (EAD1, 6 µM or 10 µM) on IFNγ and TNFα-induced cell death in murine cell lines (MC38, CT26, B16, 4T1, EMT6) at 24 h (left panel) and 48 h (right panel). Statistical significance was determined by one-way ANOVA (*p < 0.05, **p < 0.01, ***p < 0.001, ****p < 0.0001, ns = not significant, *n* = 3). **C** scRNAseq data showing expression of *Ddit4, Sesn2, Sesn3, Castor1*, and *Castor2* genes in *Trp53*^CON^ and *Trp53*^KO^ MC38 cells isolated in vivo. **D** Fold change in Castor1 mRNA levels in *Trp53*^CON^ and *Trp53*^KO^ MC38 cells treated with IFNγ, TNFα, their combination, or untreated (control) for 24 h. Statistical significance was determined by one-way ANOVA (****p < 0.0001, *n* = 3). **E** Dot plots showing expression of *CDKN1A*, *DDIT4, SESN3*, and *CASTOR1* in various WT, *TP53* mutated and truncated HCT116 cells.

Autophagy is a vital cellular process that plays a key role in suppressing cancer cell apoptosis [25, 26]. Notably, autophagy has also been shown to inhibit TNF-α-induced apoptosis, serving as a mechanism of cancer immune evasion [21, 22, 27]. Since mTORC1 is a well-established inhibitor of autophagy, p53’s role in suppressing mTOR signaling is particularly relevant [28–30]. To further investigate this relationship, we examined mTORC1 activity and autophagy in *Trp53*^KO^ and *Trp53*^CON^ MC38 cells. Our analysis revealed that *Trp53*^KO^ MC38 cells exhibit significantly higher mTORC1 activity compared to *Trp53*^CON^ MC38 cells as measured by levels of phosphorylated p70 S6 Kinase (Fig. 6A). To assess autophagy levels, we measured p62 protein, a widely used marker of autophagic activity. p62 levels were significantly elevated in *Trp53*^KO^ MC38 cells relative to *Trp53*^CON^ MC38 cells (Fig. 6A), suggesting lower autophagy activity in *Trp53*^KO^ cells. In addition, we found that inhibiting autophagy potentiates TNFα/IFNγ-induced cell death in multiple murine cancer cell lines (Fig. 6B). These findings suggest that mutant *Trp53* suppresses mTORC1 activity, leading to enhanced autophagy and ultimately inhibiting apoptosis.

Consistent with the notion, scRNA-seq analysis revealed that *Trp53*^KO^ cells exhibit reduced expression of genes involved in mTORC1 inhibition, including *Ddit4, Sesn2, Sesn3, Castor1*, and *Castor2* [29, 31–33] (Fig. 4A, B, Fig. 6C, and Supplementary Table 1, 2). Given that CASTOR1 is a known negative regulator of mTORC1 activity [34], we focused on its expression. RT-qPCR analysis confirmed that *Castor1* expression was significantly reduced in *Trp53*^KO^ MC38 cells compared to *Trp53*^CON^ MC38 cells (Fig. 6D). These data suggest that the Trp53 mutation in MC38 cells enhances the expression of mTORC1 inhibitor genes, which potentially inhibits mTORC1 signaling and promotes autophagy.

To determine whether *TP53* mutations similarly impact these genes in human cells, we analyzed a recently published scRNA-seq dataset derived from HCT116 (a human colorectal cancer cell line) engineered to harbor all known TP53 hotspot mutations [17]. The typical TP53 target gene *CDKN1A* is downregulated in all cells harboring various *TP53* mutations (Fig. 6E). Conversely, mTORC1 inhibitor genes, including *DDIT4*, *SESN3* and *CASTOR1*, displayed variable expression patterns in *TP53* mutants: their expression remained unaffected in some mutants but was completely absent in others (Fig. 6E). Notably, in the *G245S* mutant, *SESN3* and *CASTOR1* expression was modestly reduced, whereas DDIT4 expression remained largely unaffected (Fig. 6E). Collectively, these results demonstrate certain p53 mutants, including G245S (and its murine counterpart G242V), despite losing other canonical functions, retain some levels of the wild-type p53’s ability to suppress mTORC1 and enhance autophagy, thereby inhibiting responses to immunotherapy.

## Discussion

In this study, we demonstrated that deleting mutant *Trp53* in MC38 cells significantly enhances the efficacy of PD-1 immune checkpoint blockade (ICB) therapy. This improved response is driven by increased CD8⁺ T cell clonal expansion and reduced Treg infiltration within the TME. Mechanistically, *Trp53* deletion leads to hyperactivation of mTOR signaling and suppression of autophagy, sensitizing tumor cells to cytokine-induced apoptosis. These findings reveal a novel mechanism linking *Trp53* mutations to immunotherapy outcomes, providing fresh mechanistic insights into how *Trp53* status influences immune responses in cancer.

A substantial body of research, especially in lung cancer, indicates that *TP53* mutations are strongly correlated with increased expression of immune checkpoint molecules, activation of T-effector genes, and enrichment of IFNγ signature genes [7]. These findings are corroborated by multiple studies [8, 9]. Notably, non-small cell lung cancer (NSCLC) patients harboring *TP53* mutations exhibit improved clinical outcomes, including prolonged survival, in response to PD-1 inhibitor treatment [9–12]. However, a major confounding factor in these studies is the possibility that *Trp53* mutations indirectly affect tumor mutational burden (TMB) and neoantigen load, rather than exerting a direct impact on immune surveillance [35]. In contrast, some p53 mutants, including P142L, P152Q, A161V, C174Y, R175H, R248W, R249S, R273H, and R280K, have been reported to interact with TBK1 and inhibit STING activation [6], and potentially inhibit cancer immunotherapy. In addition, another study demonstrates that *Trp53* loss, within the genetic context of Kras mutation and TMB, promotes immune resistance in an autochthonous mouse lung cancer model [36]. This observation aligns with the well-established role of wild-type P53 in stimulating innate immune responses [37]. Our study uncovers distinct and direct mechanisms of the G242V mutant (corresponding to the human TP53 hotspot mutation G245S), including suppression of mTOR signaling and induction of autophagy. We also showed that this property of G245S is shared with some but not all p53 mutants. Biochemical, structural, and in vivo studies have demonstrated that the G245S *TP53* mutation retains greater residual p53 function compared to other mutations, such as R248Q. Specifically, G245S preserves approximately 25% of wild-type p53 transcriptional activity for select promoters, such as p21, while R248Q exhibits negligible transcriptional activity and fails to induce canonical targets like *PUMA* and *caspase-3* [38]. Structurally, G245S induces localized destabilization in the DNA-binding domain’s L3 loop, leading to a reduction of DNA affinity by approximately 15-fold. In contrast, R248Q disrupts both DNA minor-groove binding and global domain stability [16, 39]. In vivo, G245S/- mice display delayed tumor onset, whereas R248Q/- mice exhibit accelerated tumorigenesis, expanded stem cell pools, and reduced survival [38]. Despite its weakened oncogenic activity, the G245S p53 mutant, through its preserved capacity to suppress mTORC1, hinders the efficacy of cancer immunotherapy. Interestingly, a recent study demonstrated that the mouse R172H mutation—a well-characterized missense mutation in the p53 DNA-binding domain classified as a structural mutant that impairs p53’s tumor suppressor function and exhibits both dominant-negative and gain-of-function (GOF) effects—inhibits the antitumor immune response by upregulating the CXCL1–neutrophil axis [40]. Collectively, multiple in vitro and mouse studies demonstrate the critical role of p53 mutants in inhibiting antitumor immunity, albeit through diverse mechanisms.

Under stress conditions, p53 generally inhibits mTORC1 activity through multiple mechanisms [29, 31–33]. One key pathway involves p53-mediated activation of AMPK, which inhibits mTORC1 via the TSC1/2 complex. Additionally, p53 can also decrease S6K1 activity and promote 4E-BP1 dephosphorylation, further limiting mTORC1 signaling. P53 has also been shown to induce genes encoding proteins that regulate mTORC1 activation. p53 induces PTEN transcription, which in turn inhibits the PI3K/AKT pathway, leading to mTORC1 suppression.

Moreover, DDIT4, a transcriptional target of P53, suppresses mTORC1 through the same TSC1/2-dependent mechanism. The SESTRIN (SESN1–3) family, also transcriptionally regulated by p53, plays a crucial role in suppressing both mTORC1 and mTORC2 [41]. In our experimental system, we found no evidence of p53-mediated Pten regulation. However, we observed a significant upregulation of *Sesn2*, *Sesn3*, and *Ddit4* in MC38 cells, specifically dependent on the *Trp53*^G245S^ mutation. Furthermore, we identified CASTOR1, a known mTORC1 inhibitor, as an additional p53^G245S^-regulated gene in these cells. Consistent with these findings, we demonstrated that wild-type p53 promotes the expression of these mTORC1 regulatory genes in human HCT116 cells. Notably, different p53 mutants exhibited varying degrees of functional deficiency in this context. Specifically, the p53^G245S^ mutant retained some of its ability to induce the expression of these mTORC1 regulatory genes. These results align with recent studies reporting that p53 deletion in human lung and colon cancer cell lines, including HCT116, leads to increased mTORC1 activation. Moreover, several common p53 hotspot mutants (R175H, R248W, and R273H) failed to suppress mTORC1 activation in a p53-null background, underscoring the functional heterogeneity of p53 mutants in regulating mTORC1 activity [42].

Our study demonstrates a new mechanism by which p53 mutants promote cancer immunotherapy resistance through the modulation of mTORC1 activation and autophagy inhibition. This observation highlights the potential of targeting the P53/mTORC1 axis to improve cancer immunotherapy outcomes.

## Materials and methods

### Mice

All animal procedures and methods were performed in accordance with relevant guidelines and regulations. Animal experiments were approved by the Institutional Animal Care and Use Committee of the University of Pittsburgh (protocol number 18093244) and by the Hackensack Meridian Health Center for Discovery and Innovation Institutional Animal Care and Use Committee (protocol number AUP-25-17). Mice were maintained in a specific-pathogen-free animal facility under approved conditions. WT (stock #000664) and RAG1^-/-^ (B6.129S7-*Rag1*^*tm1Mom*^/J, 002216) mice on the C57Bl/6 J background, as well as NSG (NOD.Cg-*Prkdc*^*scid*^ *Il2rg*^*tm1Wjl*^/SzJ, 005557; RRID:BCBC_4611) were purchased from the Jackson laboratory. Male and female mice aged 6 to 12 weeks were used in the study. The sample size for each group was calculated based on a power analysis to ensure adequate statistical power.

### Tissue culture

MC38 colon adenocarcinoma cells (Cat# YC-A002, RRID:CVCL_B288) were maintained in Dubecco’s Modified Eagle Medium, high glucose supplemented with 10% fetal bovine serum in the presence of benzylpenicillin (100 U/mL), streptomycin (100 mg/mL), and 2 mmol/L L-glutamine and 1% penicillin-streptomycin. CT26 colon cancer (Cat# YC-A002, (RRID:CVCL_7254)), and 4T1 breast carcinoma cells (RRID:CVCL_0125) were all cultured in RPMI-1640 (#11875-093, Gibco) supplemented with 10% FBS and 1% penicillin-streptomycin. EMT6 breast carcinoma cells (ATCC Cat# CRL-2755, RRID:CVCL_1923) were cultured in Waymouth’s medium (#11220-035, Gibco) supplemented with 15% FBS and 1% penicillin-streptomycin. The cells were frequently checked for mycoplasma using Universal Mycoplasma Detection kit (#30-1012k, ATCC).

### Verification of G242V and S258I mutations in MC38 cells

Mutations in *Trp53* were confirmed through PCR amplification and Sanger sequencing. Genomic DNA was extracted from MC38 wild-type and clonal lines (#6 and #16) using a tissue DNA extraction kit with heat lysis at 95 °C and stabilization buffer treatment. To verify the G242V mutation, the region encompassing exon 7 was amplified using the primers mP53 Forward (5’ GCTATAGCCAGCCATTCCC 3’) and mP53 Reverse (5’ ACCATCCAATCCAATCGGACA 3’). PCR was performed using OneTaq® Quick-Load® 2X Master Mix under the following thermal conditions: 95 °C for 5 min; 35 cycles of 95 °C for 30 sec, 58 °C for 30 sec, 72 °C for 1 min; and a final extension at 72 °C for 5 min. PCR products were purified using a PCR purification kit and sequenced using the forward sequencing primer TGGTAGGTTAGGTTAGCCTGT. The G242V mutation was confirmed by identifying a G-to-T nucleotide substitution in exon 7. Similarly, the S258I mutation was validated for having a T-to-G substitution resulting in a serine-to-isoleucine amino acid change.

### Generation of *Trp53* deletion in MC38 cells

*Trp53* knockout was performed using CRISPR-Cas9. Briefly, single-guide RNA (sgRNA) was designed using online CRISPR Design Tool (https://crispr.cos.uni-heidelberg.de) and cloned into plasmid lentiCRISPRv2GFP (Addgene, catalog no. 82416). The sgRNA sequences were designed to delete exon 4 of mouse *Trp53*. They are gRNA1: ACAGCCATCACCTCACTGCA, gRNA2: ACACTCGGAGGGCTTCACTT. The plasmids were transfected into the MC38 cell line using Lipofectamine 2000 (Thermo Fisher Scientific, catalog no. 11668030). Transfected cells were sorted and single cells cloned, and mutant cells were identified using genomic DNA PCR and confirmed by Western blot analysis. The genomic target sequences used for targeting screening were AGGAAATCAGGAACTAACTCTCTGCTCTT (forward) and TGCACATAACAGACTTGGCTGTCC (reverse).

### Tumor models

Mice were shaved and inoculated intradermally with *Trp53*^CON^ and *Trp53*^KO^ MC38 cells at 10^6^ cells/50uL PBS/tumor. One tumor was inoculated per mouse. Tumor length (L) and width (W) were measured using a digital caliper. Tumor volume was calculated using the formula: L x W x W x 0.5. Tumors were treated with anti-PD-1 purchased from BioxCell *InVivo*MAb anti-mouse PD-1 (catalog #BE0146). Antibodies were aliquoted and diluted in PBS to the required concentration to inject each mouse intraperitoneally with 100ug in 100uL. Mice were treated on days 5, 9, 13, and 17, and tumors were allowed to grow up beyond the last treatment.

### Tissue processing

Mice were euthanized and tumors immediately dissected. Excess skin, hair, fat, and connective tissue were removed. Tumors were transferred into 6-well plates containing 0.25 mg/mL LiberaseTL (Roche, 5401020001) and 0.33 mg/mL DNase (Sigma-Aldrich DN25-10MG) in RPMI, minced with scissors, and digested in a tissue culture incubator for 30 min without agitation. Digestion was quenched with 3 mL RPMI. Samples were strained through 70-uM strainers and pushed through using a pestle, then strained through 30uM nylon mesh. Samples were pelleted and resuspended in 2% FBS in HANKS.

### Flow cytometry

Staining was performed in 96-well V-bottom plates. For cytokine analysis, cells were first stimulated using the Leukocyte Activation Cocktail, with BD GolgiPlug (BD Biosciences 550583) in a tissue culture incubator for 4 h. Cells were initially stained in 0.1% Ghost Dye Violet 510 (Tonbo Biosciences, 13-0870-T500) in PBS on ice for 30 min. They were then stained in antibody cocktails in 2% FBS in HANKS on ice for 5 min. Cells were then filtered through 30uM nylon mesh. Samples were run on a Cytek Aurora.

The following antibodies were used: ArgI (eBioscience, catalog #46-3697-82, clone A1exF5), CD4 (BD Biosciences, 612844, RM4-5; RRID:AB_2870166), CD8a (BD Biosciences, 566096, 53-6.7; RRID:AB_2739500), CD11b (BD Biosciences, 564443, M1/70; RRID:AB_2738811), CD11c (Biolegend, 117312, N418; RRID:AB_389328), CD24 (BioLegend, 101822, M1/69; RRID:AB_756048), CD45 (BioLegend, 103130, c30-F11; RRID:AB_893339), CD44 (BioLegend, 103005, IM7; RRID:AB_312956), CD62L(BD Biosciences, 560514; RRID:AB_10611861), CD80 (BioLegend,104724, 16-10A1; RRID:AB_2075999), CD86 (BioLegend, 105043, GL-1; RRID:AB_2566722), CD206 (BioLegend, 141708, C068C2; RRID:AB_10900231), F4/80 (BioLegend, 123130, BM8; RRID:AB_2293450), Gr-1, Ly-6C (BioLegend, 128037, HK1.4; RRID:AB_2562630), Ly-6G (BioLegend, 127628, 1A8; RRID:AB_2562567), MHCII, PD-1 (BD Biosciences, 551892, J43; RRID:AB_394284), Tim-3 (BioLegend, 119721, RMT3-23; RRID:AB_2616907), LAG-3 (BioLegend, 125212, C9B7W; RRID:AB_2561517), ST2 (eBioscience, 46-9333-82, RMST2-33), CD103 (BD Biosciences, 565849, M290; RRID:AB_2739377), TCF1 (Cell Signaling Technology, 14456, C63D9; RRID:AB_2798483), Foxp3 (BioLegend, 126410, MF-14; RRID:AB_2105047), GzmB (BioLegend, 372204, QA16A02; RRID:AB_2687028), Ki-67 (BioLegend, 652410, 16A8; RRID:AB_2562141), and IFN-γ (BioLegend, 505826, clone XMG1.2; RRID:AB_2295770). All antibodies were used at 1:100 dilution.

### scRNAseq

Dissected and trimmed tumors were washed in pre-cooled RNase-free H_2_O and transferred into MACS Tissue storage solution (Miltenyi Biotec, 130-100-008). Samples were shipped on ice overnight to Novogene. Samples were processed according to the standard 10X pipeline. Three tumors from separate mice were prepared separately for each condition. The sample with the highest best cell viability and concentration for each condition was chosen. From the two samples, 10000 cells (target 7000 cells in output) per sample were loaded onto two separate lanes of a Chromium Chip in a Chromium Controller (10X Genomics) with 5’ chemistry for gene expression and TCR analysis. Libraries were sequenced on an Illumina Novaseq 6000 S4 with 30000 reads/cell ( ~ 200 M reads total, 60 Gb data output).

Cellranger 2 was used to align fastq files, which were aligned to the mm10 reference genome and generate barcode, feature, and count matrices. Initial downstream analysis was performed using Seurat and Scanpy. Custom code was used for further analyses.

### Cell death stimulation and autophagy inhibition

MC38 and *Trp53*^KO^ MC38 clones (each 8 × 10^4^ cells per well) were seeded in 24-well plates for 24 h before treatment with murine IFNγ (50 ng/ml, #315-05, Pepro Tech), murine TNFα (50 ng/ml, #315-01 A, Pepro Tech), their combination, or left untreated. The cytokines were added into each well, and the cells were incubated at 37 °C for 24 h and 48 h prior to counting with a TC20 cell counter (Bio-Rad). For autophagy inhibition, MC38, B16, 4T1, EMT6 cells (8 × 10^4^ per well), and CT26 cells (10^5^ per well) were seeded in 24-well plates. Next day, the cells were treated with 50 ng/ml of both IFNγ and TNFα as well as 6 µM and/or 10 µM of EAD1 (#S8576, Selleck Chemicals). The cells were then incubated at 37 °C for 24 h and 48 h prior to counting with a TC20 cell counter (Bio-Rad).

### Western blot analysis

*Trp53*^CON^ and *Trp53*^KO^ MC38 clone were treated with both IFNγ and TNFα or left untreated and incubated for 24 h, 36 h, and 48 h at 37 °C. The harvested cells were lysed with lysis buffer and ran into Mini-Protean TGX pre-cast gels (#4561085, Bio-Rad) prior to transferring to PVDF membranes using a Trans-Blot Turbo (#1704150, Bio-Rad). The membranes were then blocked with 6% non-fat milk and subjected to primary antibodies for overnight incubation. The mouse primary antibodies used for the experiments include caspase 3 (#9662; RRID:AB_331439), caspase 8 (#4927; RRID:AB_2068301), cleaved caspase 3 (#9664; RRID:AB_2070042), cleaved caspase 8 (#8592; RRID:AB_10891784), p62 (#39749; RRID:AB_2799160), phosphorylated P70S6K (#9205; RRID:AB_330944) and beta-actin (#4970; RRID:AB_2223172). The membranes were incubated with secondary antibody, mouse anti rabbit IgG (#93702; RRID:AB_2800208) after and before washing steps. All antibodies were purchased from Cell Signaling (Danvers, MA, USA). All washing steps were conducted with TBST at room temperature. At the end, an equal amount of SuperSignal West Pico Plus (#34580, Thermo Scientific) was added to the membranes to visualize the bands in a ChemiDoc imaging system (Bio-Rad). The bands were quantified with Image Lab version 6.1 (Bio-Rad).

### Quantitative RT-PCR

The cells were treated with mouse IFNγ (50 ng/ml), TNFα (50 ng/ml), their combination or left untreated for 24 h. Total RNA was isolated from the cells using RNeasy Plus Mini kit (#74134, Qiagen). Complementary DNA was synthesized using qScript cDNA Synthesis kit (#95047, Quanta bio). Quantitative RT-PCR was performed using PowerUp SYBR Green Master Mix (#A25742, Applied Biosystems) to detect *Castor1* gene with the following primers: CAGAACCGCTTTTGTGTCCTCAC (forward), GGAGAAAGCGAAGAACGGAATGG (reverse). GAPDH was utilized as endogenous control using AGGTCGGTGTGAACGGATTTG (forward) and GGGGTCGTTGATGGCAACA (reverse) primers. The MicroAmp Optical 96-well Reaction plates (#4306737, Applied Biosystems) were run in a QuantStudio 5 (Applied Biosystems).

### Statistical analyses

GraphPad Prism 6.0 software package (GraphPad Software, Inc.; RRID:SCR_002798) was used for the analysis. Statistical analysis was done using the Student *t* test (two-tailed), ordinary one-way ANOVA, or the log-rank survival analysis. A *P* value of <0.05 was considered to be statistically significant. No statistical correction was used.

## Supplementary information

Supplemental Figures

Original Data-WB

Original Data-qpcr

Supplemental Table 1

Supplemental Table 2

## Data Availability

The generated data is available upon request from the corresponding author. Public scRNA-seq data were retrieved from EMBL BioStudies (https://www.ebi.ac.uk/biostudies/), under accession number E-MTAB-13904. The single-cell RNA-sequencing data generated in this study are publicly available through the Gene Expression Omnibus under accession number GSE295668. (RRID:SCR_005012).
