# Supplementary material for: mTORC1 suppression by Trp53 mutation drives resistance to immune checkpoint blockade
Source: Cell Death Dis. 2026 Jul 7;17(1):841. doi: 10.1038/s41419-026-09067-4 (PMC13620114; doi:10.1038/s41419-026-09067-4)
Supplement: Supplementary file 1 [file 41419_2026_9067_MOESM1_ESM.pdf]

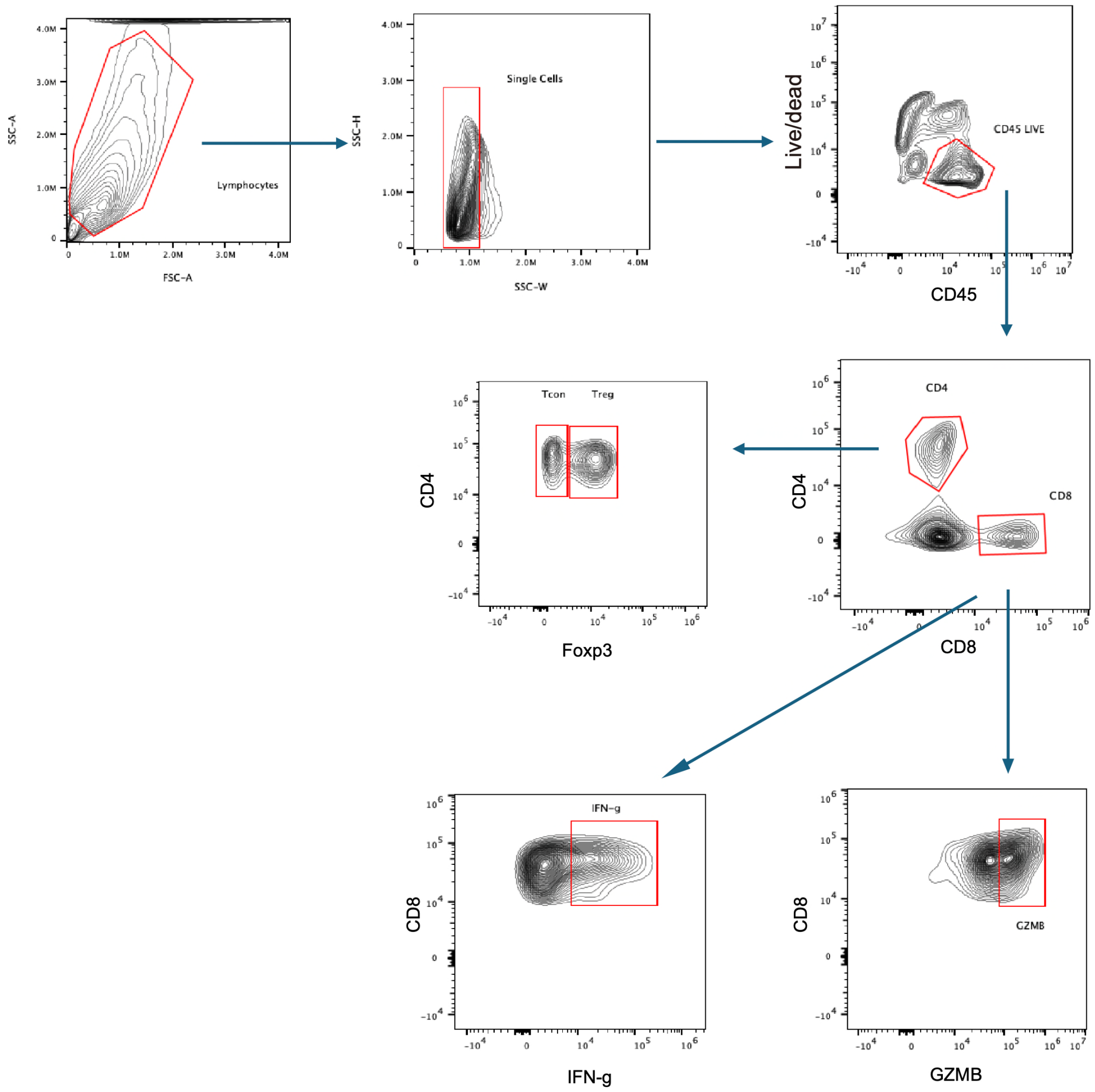

Supplemental Figure 1. Gating strategy for T cells. Live CD45+ single cells were further gated on CD4 and CD8 markers. Treg populations were gated as Foxp3+ CD4+ T cells. IFNG and GzmB expression were further gated in CD8+ T cells.

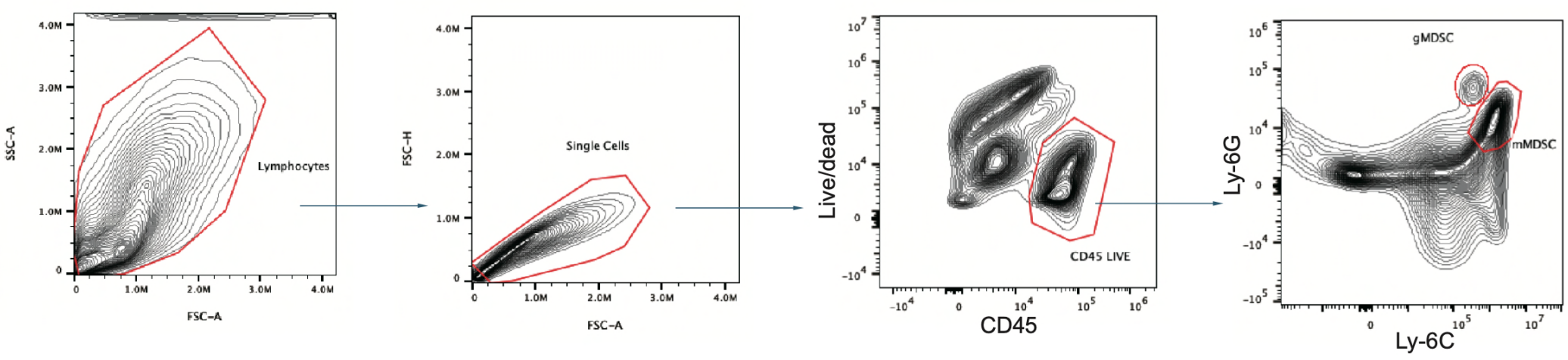

Supplemental Figure 2. Gating strategy for MDSC. Live CD45+ single cells were further gated on monocytic and neutrophilic MDSC markers Ly6c and Ly6g as indicated.
