# Supplementary material for: mTORC1 suppression by Trp53 mutation drives resistance to immune checkpoint blockade
Source: Cell Death Dis. 2026 Jul 7;17(1):841. doi: 10.1038/s41419-026-09067-4 (PMC13620114; doi:10.1038/s41419-026-09067-4)
Supplement: Supplementary file 2 [file 41419_2026_9067_MOESM2_ESM.pdf]

Figure 1A

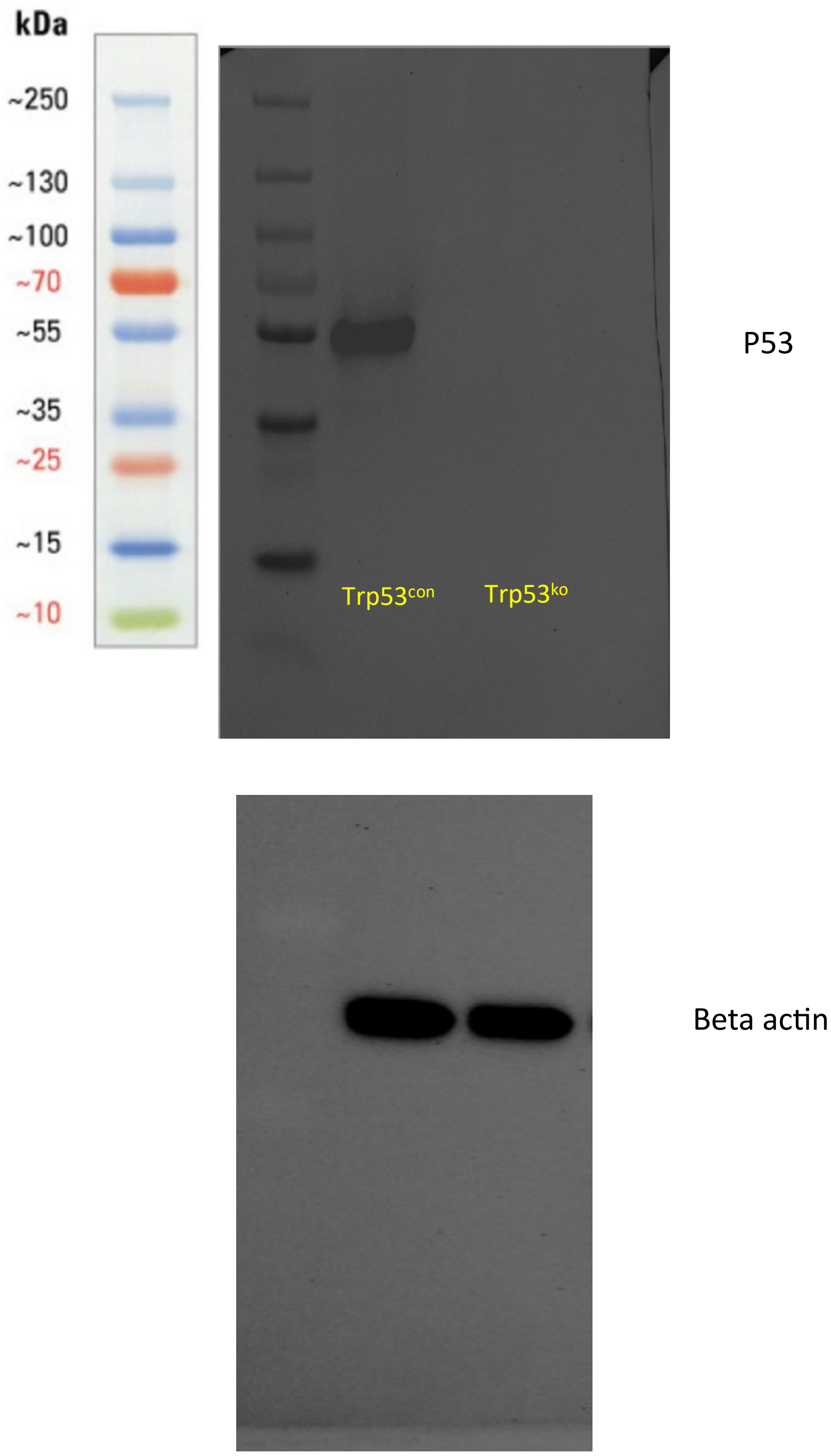
kDa
~250
~130
~100
~70
~55
~35
~25
~15
~10
Trp53con
Trp53ko
P53
Beta actin

Fig 5B

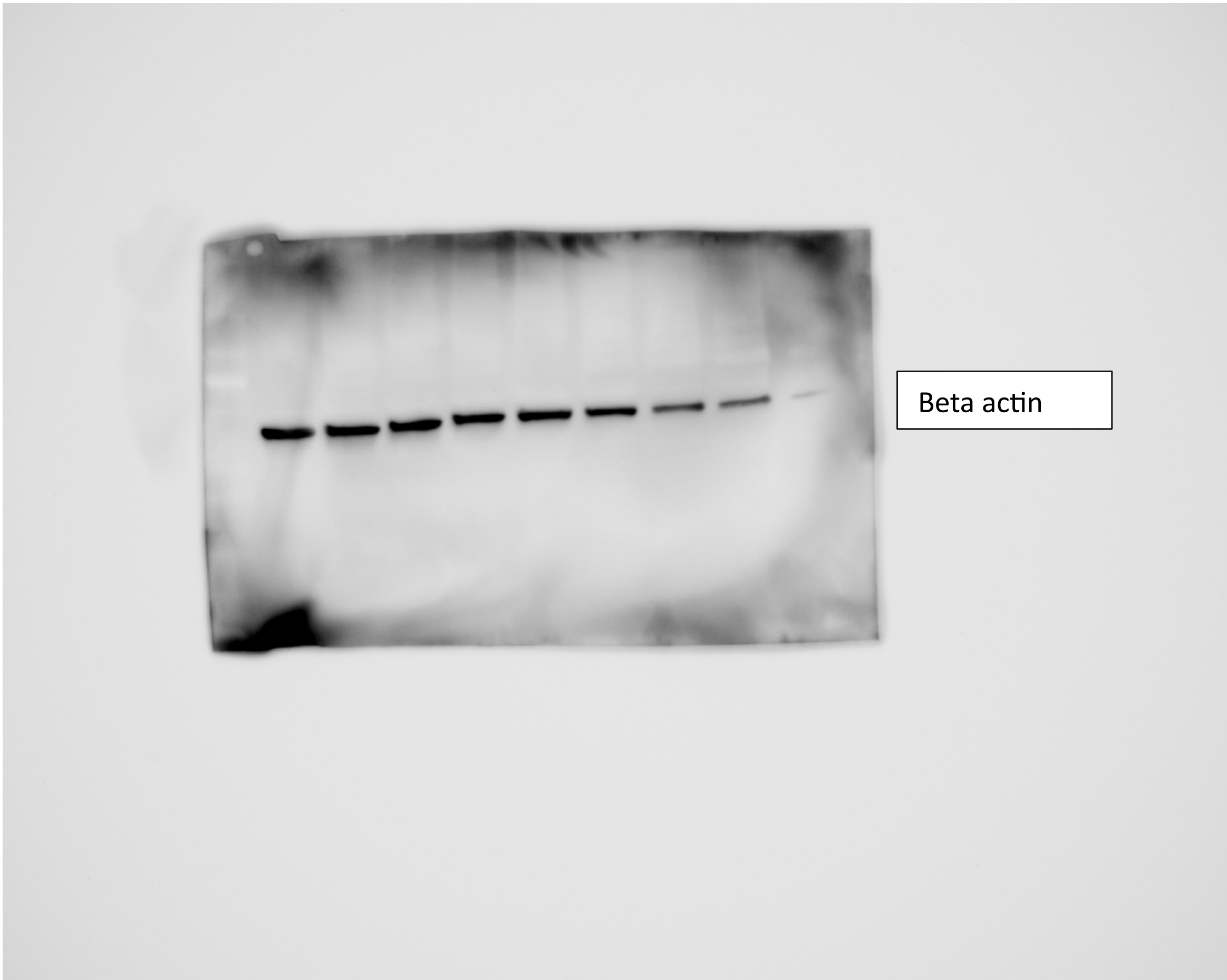

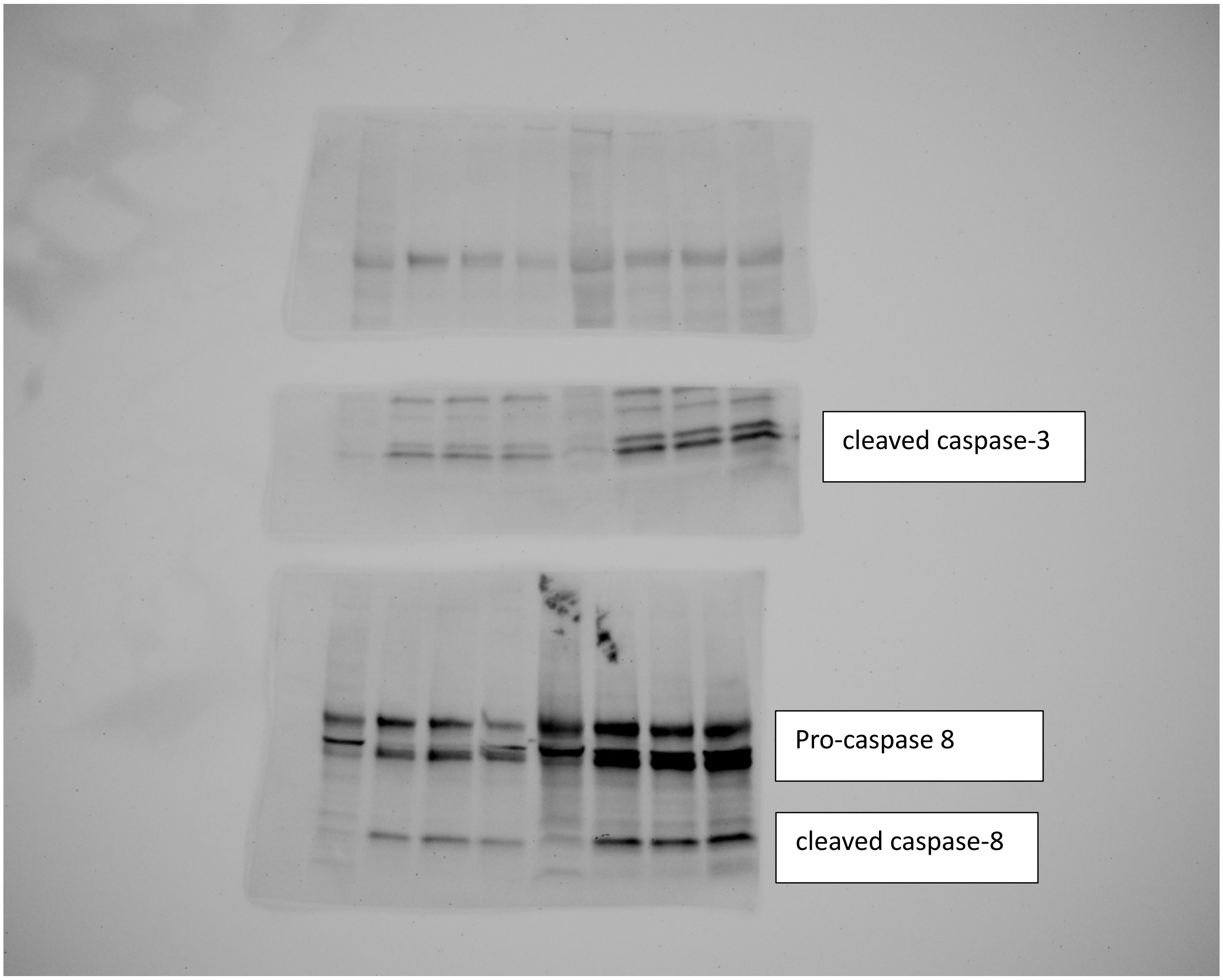
cleaved caspase-3
Pro-caspase 8
cleaved caspase-8

Fig 6A

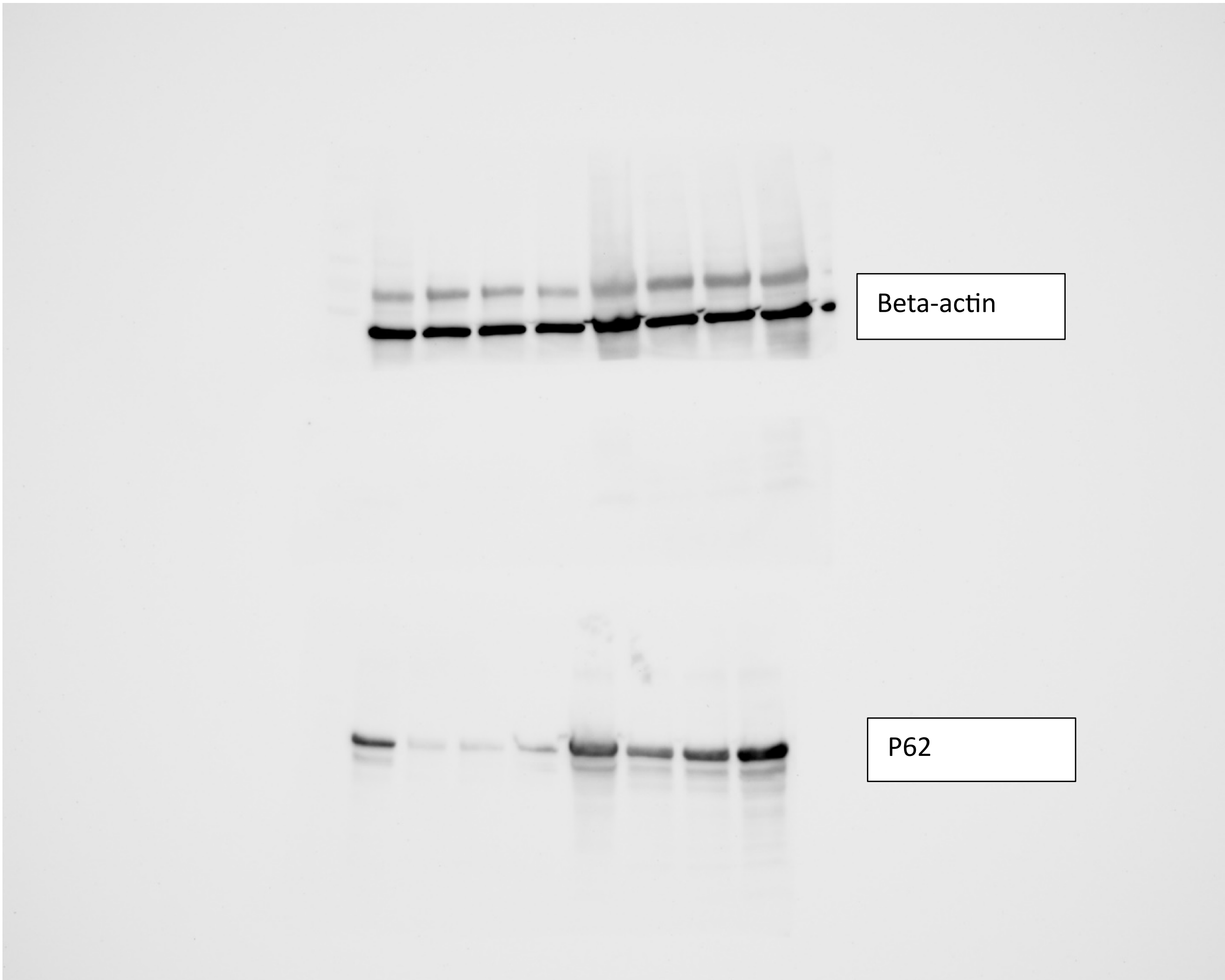

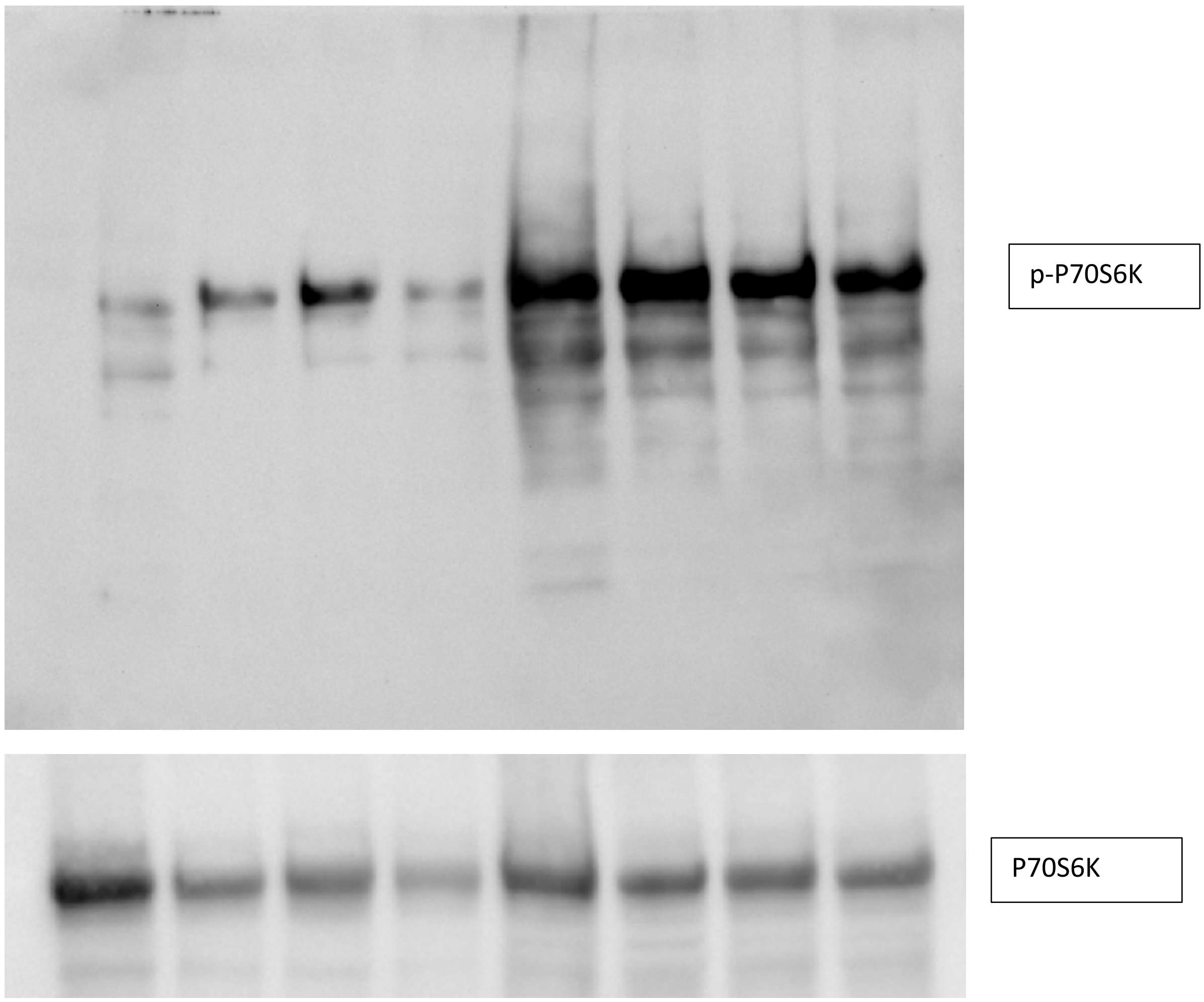
p-P70S6K
P70S6K
